# Enzymatic hydrolysis processing of soybean meal altered its structure and *in vitro* protein digestive dynamics in pigs

**DOI:** 10.3389/fvets.2024.1503817

**Published:** 2024-11-22

**Authors:** Dapeng Wang, Honglin Du, Xiuquan Dang, Yufei Zhao, Jiaxuan Zhang, Rujie Liu, Zhenying Ge, Qingzhen Zhong, Zewei Sun

**Affiliations:** Key Laboratory of Animal Production, Product Quality and Security, Ministry of Education, Jilin Province Key Laboratory of Animal Nutrition and Feed Science, College of Animal Science and Technology, Jilin Agricultural University, Changchun, China

**Keywords:** enzymatic hydrolysis, soybean meal, protein, structure, digestive dynamics

## Abstract

**Introduction:**

The study evaluated the enzymatic hydrolysis processing on physicochemical properties and protein digestive dynamics of soybean meal (SBM), as well as the relationship between protein secondary structure and digestive parameters was established.

**Methods:**

Scanning Electron Microscopy (SEM) and Fourier Transform Infrared Spectroscopy (FTIR) were employed to analyze the microstructure and protein structure of the SBM and enzymatic hydrolysis processed soybean meal (ESBM). SBM and ESBM were incubated with pepsin at pH 3.5 and 39°C for 30 min, then with pancreatin at pH 6.8 for 0–240 min. The *in vitro* protein digestive dynamics were described as the release dynamics of amino acids and low molecular weight peptides (AA_LMW).

**Results:**

The results showed that enzymatic hydrolysis processing did not alter the chemical composition of SBM, but changed its microstructure and protein structure. After enzymatic hydrolysis processing, the size of blocky structures of SBM decreased, exhibiting a fibrous surface and a relatively loose internal structure. The β-sheet content of ESBM was lower than that of SBM (*p* < 0.05), while the α-helix, β-turn, and α-helix/β-sheet content was higher than that of SBM (*p* < 0.05). The release rates (k) of AA_LMW in SBM and ESBM were 0.0123 min^−1^ and 0.0733 min^−1^, respectively. Enzymatic hydrolysis processing increased the CP_fast_ content of SBM (*p* < 0.05) and decreased the CP_slow_ and CP_resistant_ contents (*p* < 0.05). α-helix, β-turn, and the ratio of α-helix to β-sheet were positively correlated with CP_fast_ and k (*p* < 0.05) and were negatively correlated with CP_slow_ and CP_resistant_ (*p* < 0.05). β-sheet was negatively correlated with CP_fast_ and k (*p* < 0.05) and was positively correlated with CP_slow_ and CP_resistant_ (*p* < 0.05).

**Discussion:**

Enzymatic hydrolysis processing altered the digestive dynamics of SBM, increased the CP_fast_ content and the release rate of AA_LMW, which might be attributed to the structure changes of SBM.

## 1 Introduction

Dietary protein plays a critical role in animal production as a major feed component. In current feed evaluation systems, the nutritional value of protein ingredients in diets for pigs is typically evaluated based on the content of essential amino acids and their ileal digestibility (1, 2). Considering factors such as convenience and cost, researchers have also explored *in vitro* digestion methods to simulate and predict the ileal digestibility of amino acids and proteins in pigs, providing a rapid evaluation of the value of protein sources (3). However, these data do not explain the dynamic process of protein digestion along the gastrointestinal tract.

Protein sources with similar digestibility may exhibit distinct protein digestive dynamics, thereby affecting the postprandial absorption and metabolism of the end products of protein digestion. For instance, although both casein and whey proteins are considered highly digestible in humans (3), the rate and extent of the increase in amino acids and peptides in plasma exhibited significant differences (4). The casein group showed a slow and sustained postprandial increase of amino acids and peptides. In contrast, the whey protein group showed a rapid but transient postprandial increase of amino acids and peptides in plasma. Based on the rate and the extent of the postprandial increase of plasma AAs and peptides, Boirie et al. (4) categorized protein sources into fast protein and slow protein. Recently, Mai Anh Ton Nu et al. (5) further categorized protein sources into fast, slow, and resistant proteins based on the extent of *in vitro* protein digestion over different periods. The rate of protein digestion affects the deposition of body protein (4, 6). The slow protein can reduce body weight loss and conserve mobilization of body protein in sows (7), increase litter weight gain in piglets, and improve protein efficiency during lactation (8). Additionally, the synchrony of starch and protein digestion also affects energy and protein utilization efficiency in pigs (9). The protein digestive dynamics can be affected by the chemical composition, the protein classification, and the physicochemical properties of the protein sources (10). Different physical, chemical, and enzymatic methods can be used to modify the structure and physicochemical properties of protein sources.

Soybean meal (SBM) is the most common protein source in pig diets (11). However, SBM, a byproduct of oil extraction from soybean, also contains residual anti-nutritional factors that limit its use in piglet diets (12–15). To improve the utilization efficiency of SBM in pigs and reduce the use limitations, various processing methods such as extrusion (16), enzymatic hydrolysis (17), and fermentation (18) have been studied. These processing methods alter the physical structure of SBM, reduce their anti-nutritional factor content, and increase protein utilization efficiency in pigs. Additionally, the reduction of undigested protein entering the hindgut helps regulate hindgut pH and improve gut health (19, 20). The structure of protein influences the accessibility of enzymes for protein digestion. Some commonly used feed ingredients in pig diets exhibit structural constraints in their natural protein composition (21). Some studies have found that the secondary structure of proteins can influence both the rate and extent of protein digestion. Protein sources with a high proportion of α-helix or a high ratio of α-helix to β-sheets structures tend to have faster digestion rates and higher digestibility within the same timeframe (22–24).

Understanding the changes in structure and protein digestive dynamics of protein sources after processing can guide the optimal processing of protein sources, thereby further improving the utilization efficiency of protein by pigs. These could provide important references for evaluating the nutritional value of ingredients and their application in pig production. Therefore, we selected SBM as the research subjects in the present study, which aimed to investigate how enzymatic hydrolysis processing affects its physicochemical properties and the protein digestive dynamics represented by the release dynamics of amino acids and low molecular weight peptides (AA_LMW). Furthermore, a correlation between dynamic parameters and the protein secondary structure was established. We hypothesized that enzymatic hydrolysis processing alters the microstructure and molecular structure of the SBM, thereby affecting the protein digestive dynamics of SBM.

## 2 Materials and methods

### 2.1 Materials

SBM and enzymatic hydrolysis processed soybean meal (ESBM; K-Protein) were provided by Liaoning Complete Biotechnology Co. (Liaoning, China). The ESBM was produced using a combination of asynchronous enzymatic hydrolysis methods. Briefly, enzymatic hydrolysis processing steps were as follows: (1) SBM was ground and mixed using water to 50–60% moisture content, including an enzyme mixture (Proteases, Liaoning Province Feed Pre-digested Technical Innovation Center, Liaoning, China) as a processing aid; (2) SBM underwent enzymatic hydrolysis in the enzymatic hydrolysis tank at approximately 50°C for 6 h. No other method was used to control the pH, and the pH value range remained within 5.5–6.5 during the processing; (3) then the enzyme mixture was inactivated at temperature ≥95°C for 10–15 min; (4) drying and cooling to stabilize the product with moisture ≤12%. Three samples were randomly collected from SBM and ESBM and ground by a hammer mill through a 1 mm sieve for subsequent analysis with three replicates per analysis. Sub-samples were collected and stored at 4°C. To eliminate the interference of sample sources on the test results, the SBM before and after the enzymatic hydrolysis treatment were the same source and the same batch. Meanwhile, to improve the representativeness of the samples, the newly produced SBM, purchased in bulk by typical enzymatic hydrolysis processing enterprises, was chosen as the research object.

### 2.2 Scanning electron microscopy (SEM)

A SEM (ZEISS GeminiSEM500, Oberkochen, Germany) was employed to observe the microstructure of SBM and ESBM samples at an acceleration voltage of 2 kV. Before SEM, the samples were glued to the sample loading table, and gold was sprayed on the surface. The software Image J (Image J 1.53e, National Institutes of Health, USA) was employed to analyze the particle area of blocky structures.

### 2.3 Fourier transform infrared spectroscopy (FTIR)

The spectral data of samples were obtained using the FTIR spectroscopy (ALPHA II, Bruker, Germany). Raw spectra were recorded in the 4,000–400 cm^−1^ spectral range with 32 co-added scans at a resolution of 4 cm^−1^. Each sample was analyzed in triplicate. The software OMNIC (OMNIC 8.2, Thermo Nicolet Corp., Madison, USA) was employed to analyze the spectral data. The software Peakfit (Systat PeakFit 4.12, SeaSolve Software Inc., CA, USA) was utilized to correct and analyze each spectrum and to determine the relative content of the protein secondary structures as indicated by the following bands: α-helix (1,650–1,660 cm^−1^), β-sheet (1,600–1,640 cm^−1^), β-turn (1,660–1,670 cm^−1^), and random coil (1,640–1,650 cm^−1^) (25).

### 2.4 *In vitro* protein digestive dynamics

The *in vitro* protein digestion method was modified based on the method of Boisen and Fernandez (3, 10). For the simulation of protein digestion in the stomach, 1.0 g samples and five 6 mm diameter glass beads were placed into a 50 mL plastic centrifuge tube, with three replicates for each sample at each time point. Then, 10 mL of porcine pepsin solution (1 mg/mL P7000 Sigma) was added. The pH was adjusted to 3.5 with 1 M HCl or 1 M NaOH, and each centrifuge tube was placed in the Electric Water Bath Shaker at 39°C under continuous stirring. The incubation time with pepsin was 30 min. After incubating with pepsin for 30 min, adjust the solution to pH 6.8 using 0.2 M NaOH. The protein digestion in the small intestine was simulated by adding 10 mL of porcine pancreatin phosphate buffer solution (pH 6.8, 5 mg/mL, Sigma P7545). The incubation with pancreatin was continued in the Electric Water Bath Shaker at 39°C under continuous stirring. The incubation times with pancreatin were 0, 15, 30, 60, 90, 120, 180, and 240 min, respectively.

At each time point, the centrifuge tubes with samples were taken out and immediately placed in a −20°C refrigerator for 20 min to cool, and then centrifuged (10 min, 8,000 rpm, 4°C) to separate the insoluble protein fraction (IPF) and the soluble protein fraction (SPF). After centrifugation, the soluble fraction (supernatant) was transferred to a 50 mL volumetric flask and diluted with de-mineralized water to 50 mL. 10 mL of the diluted soluble fraction were retained for nitrogen (N) content analysis to calculate the N solubility at each digestion time point. 8 mL of the diluted soluble fraction was taken and mixed with 2 mL of 20% sulfosalicylic acid. Invert to mix thoroughly, then centrifuge (10 min, 8,000 rpm, 4°C) to separate amino acids and soluble low molecular weight peptides (AA_LMW) and soluble high molecular weight peptides (HMW). The supernatant was retained for N content analysis to calculate the AA_LMW.

### 2.5 Chemical analysis

All chemical analyses were conducted following standard laboratory methods. Determinations included analysis of dry matter (DM) (26), crude protein (CP) (27), ether extract (EE) (28), and ash (29).

### 2.6 Calculation

The N Solubility was calculated by equation 1:


(1) $$N\;\text{Solubility}\;\left(\%\right)=\frac{N_{SPF}}{N_{\text{sample}}}\times 100\%$$

Where N_sample_ (mg) is the amount of N in 1 g of sample, and N_SPF_ (mg) is the amount of N in the SPF during the *in vitro* digestion with pepsin and pancreatin.

The N content in AA_LMW was calculated by equation 2:


(2) $$\text{AA}\;\text{and}\;LMW\;\text{fraction}\;\left(\%\right)=\frac{N_{\text{AA}\_LMW}}{N_{\text{sample}}}\times 100\%$$

Where N_sample_ (mg) is the amount of N in 1 g of sample, and N_AA_LMW_ is the amount of N in the AA_LMW during the *in vitro* digestion with pepsin and pancreatin.

The N content in HMW was calculated by equation 3:


(3) $$HMW\;\text{fraction}\;\left(\%\right)=\frac{N_{SPF}-N_{\text{AA}\_LMW}}{N_{\text{sample}}}\times 100\%$$

Where N_sample_ (mg) is the amount of N in 1 g of sample, N_SPF_ (mg) is the amount of N in the SPF during the *in vitro* digestion with pepsin and pancreatin, and N_AA_LMW_ is the amount of N in the AA_LMW during the *in vitro* digestion with pepsin and pancreatin.

The N content in IPF was calculated by equation 4:


(4) $$IPF\;\text{fraction}\;\left(\%\right)=\frac{N_{\text{sample}}-N_{SPF}}{N_{\text{sample}}}\times 100\%$$

Where N_sample_ (mg) is the amount of N in 1 g of sample, and N_SPF_ (mg) is the amount of N in the SPF during the *in vitro* digestion with pepsin and pancreatin.

The dynamics of AA_LMW for SBM and ESBM during the incubations were described by an exponential equation 5 (30):


(5) $$D_{t}=D_{0}+\Delta D\times \left(1-e^{-kt}\right)$$

Where D_t_ (%) is the N content in AA_LMW at incubation time t (min), D_0_ (%) is the N content in the AA_LMW at 0 min after adding pancreatin, ΔD (%) is the maximum N content in the AA_LMW (asymptotic line), and k is the rate constant.

Based on the N content of AA_LMW during *in vitro* digestion, the protein fractions of ingredients were classified into fast protein (CP_fast_), slow protein (CP_slow_), and resistant protein (CP_resistance_), where CP_fast_ and CP_slow_ correspond to the amount of CP digested within the first 30 min and between 30 and 240 min, respectively, and CP_resistance_ = 100 − CP_fast_ − CP_slow_.

### 2.7 Statistical analysis

Statistical analysis was performed using SPSS statistical software (SPSS 26.0, IBM Corp., Armonk, NY, USA), with the Independent-Samples T-test used to analyze the data presented as mean ± standard deviation. Correlation analysis was used to analyze the relationship between protein secondary structure, protein classification, and *in vitro* protein digestive dynamics parameters. Heat graphs were generated using Origin software (Origin 2022, OriginLab Corp., Northampton, MA, USA). The level of significance was determined as *p* < 0.05.

## 3 Results

### 3.1 Chemical composition

The chemical composition of SBM and ESBM are presented in Table 1. The DM content of ESBM was significantly higher than that of SBM (*p* < 0.05), while the CP, EE, and ash contents (DM basis) did not exhibit significant differences between SBM and ESBM (*p* > 0.05).

**Table 1:** Effect of enzymatic hydrolysis processing on the chemical composition of SBM (DM basis %).

Items	SBM	ESBM
Dry matter	90.13 ± 0.05^a^	91.58 ± 0.04^b^
CP	50.40 ± 0.14	49.65 ± 0.34
EE	1.43 ± 0.33	1.27 ± 0.24
Ash	6.86 ± 0.04	6.95 ± 0.02

Means with the different letters in the same row are significantly different (*p* < 0.05).

### 3.2 Microstructural characterization

**Figure 1:**
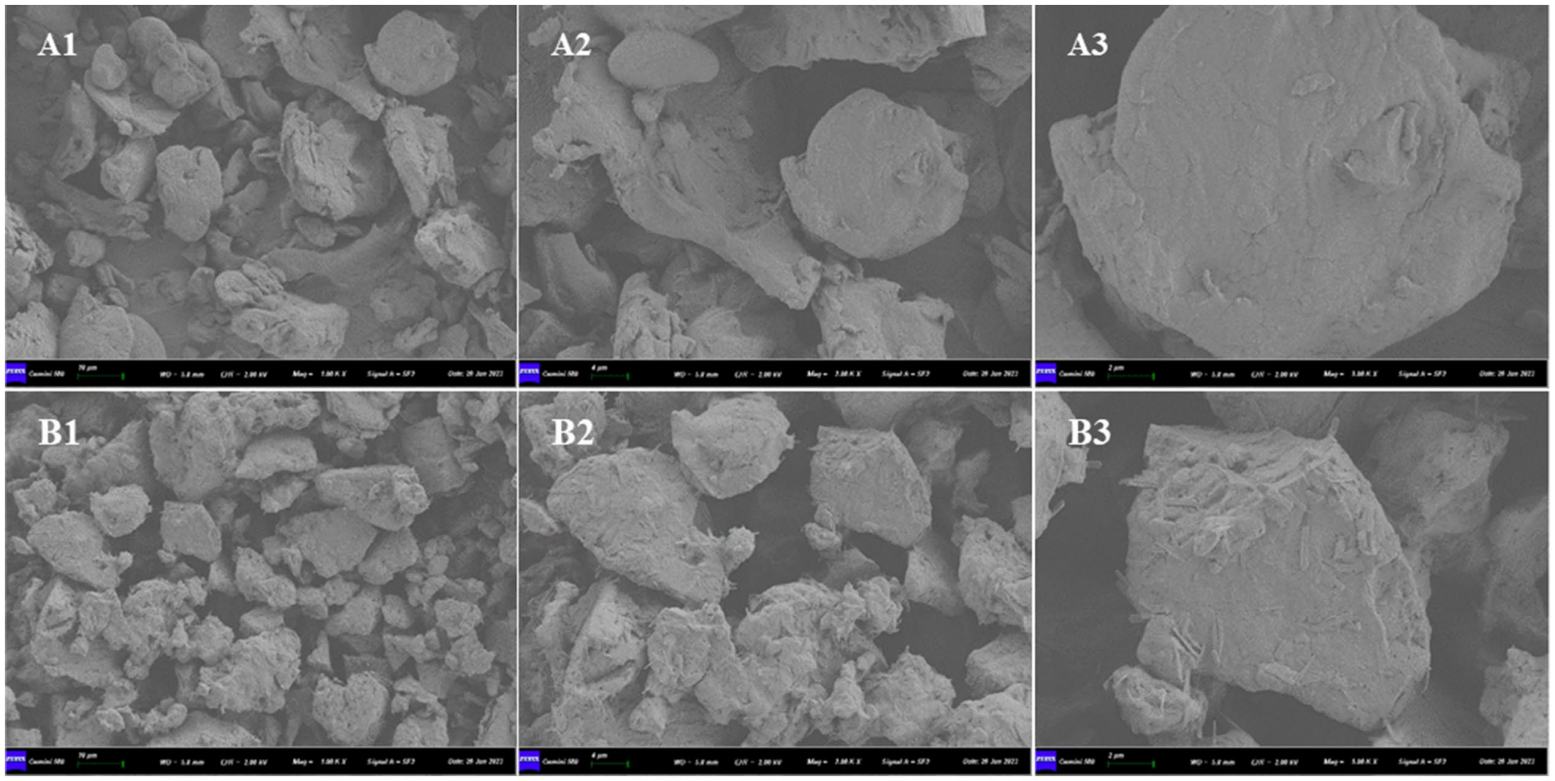
Scanning electron microscopy: **(A)** SBM and **(B)** ESBM, **(1)** magnification: 1,000×, **(2)** magnification: 2,000×, **(3)** magnification: 5,000×.

The SEM images of SBM and ESBM are presented in Figure 1. The internal structure of SBM was relatively compact, primarily comprising spherical, large blocky, and flaky structures. Following enzymatic hydrolysis processing, the morphological structure of SBM changed, and the blocky structures decreased in size (Particle area μm^2^: SBM 236.0 ± 137.9^b^; ESBM 103.6 ± 81.0^a^; *p* < 0.05), exhibiting a fibrous surface and a relatively loose internal structure.

### 3.3 Infrared spectra of protein sources

**Figure 2:**
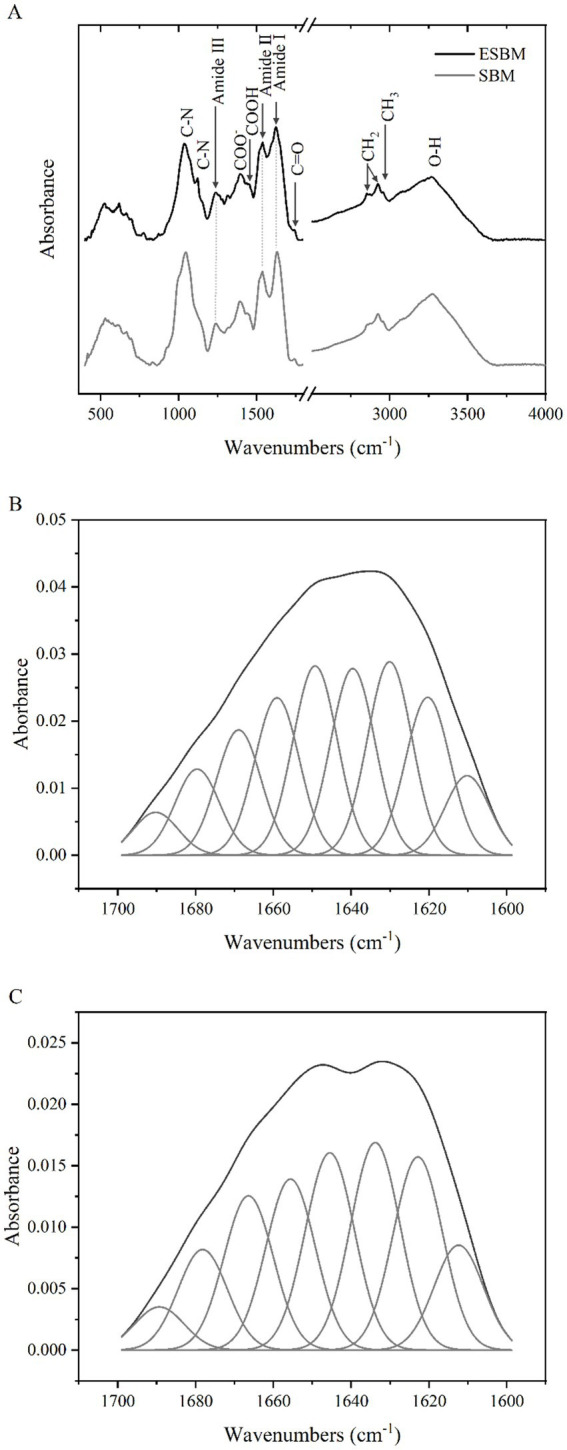
FTIR absorption spectra of SBM and ESBM **(A)**, Curve fitting of amide I band spectra of SBM **(B)**, Curve fitting of amide I band spectra of ESBM **(C)**.

The FTIR absorption spectra of the SBM and ESBM are presented in Figure 2A. After the enzymatic hydrolysis processing, the position of the Amide I band peaks (1,700–1,600 cm^−1^) in ESBM shifted toward shorter wavenumbers, and the intensity of the absorption peaks of the Amide I and II bands in ESBM decreased. The analysis results are shown in Table 2 and Figures 2B,C. The β-sheet content of ESBM was significantly lower than that of SBM (*p* < 0.05), whereas the α-helix, β-turn, or the ratio of α-helix to β-sheet content of ESBM was significantly higher than that of SBM (*p* < 0.05).

**Table 2:** The secondary structures of SBM and ESBM (%).

Item	SBM	ESBM
α-helix	12.86 ± 0.09^a^	14.96 ± 0.46^b^
β-sheet	51.04 ± 0.59^b^	42.17 ± 1.46^a^
β-turn	20.63 ± 0.22^a^	26.27 ± 1.72^b^
Random coil	15.47 ± 0.37	16.60 ± 0.73
α-helix/β-sheet	25.19 ± 0.46^a^	35.53 ± 2.36^b^

Means with the different letters in the same row are significantly different (*p* < 0.05).

### 3.4 Separation of soluble N into different molecular weight fractions

Table 3 presents the distribution of N into different molecular weight fractions. During *in vitro* digestion at 0, 15, 30, 120, 180, and 240 min, no significant differences were observed in N solubility between SBM and ESBM (*p* > 0.05). At 60 and 90 min of *in vitro* digestion, the N solubility of ESBM was, respectively, 9.8 and 8.1% higher than that of SBM (*p* < 0.05). Notably throughout all stages of *in vitro* protein digestion from 0 to 240 min, the N present in the AA_LMW of ESBM was significantly higher than that in SBM (*p* < 0.05), while the N present in the HMW was significantly lower compared to SBM (*p* < 0.05). During this process, the N solubility of SBM increased from an average of approximately 47% to about 80% within the first 30 min. At the end of pancreatin incubation (240 min), the N solubility of SBM reached about 82%, with about 54% attributed to AA_LMW and about 28% to HMW fractions, respectively. Similarly, for ESBM during *in vitro* digestion, N solubility rose from an average initial value of approximately 52% to about 81% within the first 30 min. At the end of pancreatin incubation (240 min), the ESBM showed approximately 90% N solubility, of which about 79% was present in AA_LMW and about 11% in HMW. Both SBM and ESBM exhibited a higher proportion of N present in the AA_LMW compared to the HMW fraction at the end of pancreatin incubation.

**Table 3:** N solubility and N present in the AA_LMW, HMW, and IPF as a proportion (%) of total N of SBM and ESBM at different time points during the sequential incubation with pancreatin.

Incubation times	N solubility		AA_LMW		HMW		IPF	
	SBM	ESBM	SBM	ESBM	SBM	ESBM	SBM	ESBM
0	46.6 ± 4.2	52.3 ± 1.6	26.2 ± 2.1^a^	50.1 ± 1.8^b^	20.3 ± 5.2^b^	2.2 ± 0.3^a^	53.4 ± 4.2	47.7 ± 1.6
15	78.9 ± 2.6	75.7 ± 1.2	28.7 ± 3.0^a^	61.3 ± 1.6^b^	50.2 ± 4.5^b^	14.3 ± 1.9^a^	21.1 ± 2.6	24.3 ± 1.2
30	80.4 ± 4.2	81.3 ± 3.5	36.2 ± 1.2^a^	64.3 ± 3.1^b^	44.2 ± 4.3^b^	17.0 ± 1.0^a^	19.6 ± 4.2	18.7 ± 3.5
60	79.5 ± 2.1^a^	89.3 ± 2.0^b^	43.6 ± 2.5^a^	66.7 ± 1.6^b^	36.0 ± 4.6^b^	22.6 ± 1.5^a^	20.5 ± 2.1^b^	10.7 ± 2.0^a^
90	84.1 ± 1.2^a^	92.2 ± 2.8^b^	47.2 ± 1.6^a^	63.5 ± 2.4^b^	36.8 ± 2.5^b^	28.7 ± 1.7^a^	15.9 ± 1.2^b^	7.8 ± 2.8^a^
120	87.3 ± 3.1	87.7 ± 3.3	50.9 ± 2.2^a^	69.4 ± 3.0^b^	36.4 ± 3.5^b^	18.4 ± 1.3^a^	12.7 ± 3.1	12.3 ± 3.3
180	86.3 ± 5.2	88.4 ± 3.4	51.4 ± 3.3^a^	78.4 ± 3.3^b^	34.9 ± 6.4^b^	10.0 ± 0.7^a^	13.7 ± 5.2	11.6 ± 3.4
240	82.3 ± 3.5	89.9 ± 5.1	53.9 ± 1.8^a^	78.8 ± 4.2^b^	28.4 ± 4.2^b^	11.2 ± 1.3^a^	17.7 ± 3.5	10.1 ± 5.1

Within the same molecular weight fraction, means with the different letters in the same row are significantly different (*p* < 0.05).

Enzymatic hydrolysis processing correspondingly resulted in a significant increase in the CP_fast_ content of SBM (*p* < 0.05), while concurrently leading to a significant reduction in both the contents of CP_slow_ and CP_resistant_ (*p* < 0.05) (Table 4).

**Table 4:** Effect of enzymatic hydrolysis processing on *in vitro* protein digestive dynamics of SBM.

	SBM	ESBM
Protein classification based on digestion rate		
CP_fast_, % of total CP	36.2 ± 1.2^a^	64.3 ± 3.1^b^
CP_slow_, % of total CP	17.7 ± 0.6^b^	14.4 ± 1.1^a^
CP_resistant_, % of total CP	46.1 ± 1.8^b^	21.2 ± 4.2^a^
Dynamics parameter estimates		
D_0_, %	25.24 ± 2.22^a^	50.15 ± 1.68^b^
ΔD, %	33.38 ± 1.12^b^	16.42 ± 1.05^a^
k, min^−1^	0.0123 ± 0.0000^a^	0.0733 ± 0.0058^b^

Means with the different letters in the same row are significantly different (*p* < 0.05).

### 3.5 Release dynamics of the N present in the AA_LMW

The N content in the AA_LMW fraction increased rapidly during the initial 120 min of *in vitro* digestion. Subsequently, the release rate relatively stabilized. Therefore, the N release dynamics parameters of AA_LMW were calculated based on data from the first 120 min. The initial N present in the AA_LMW D_0_ and the release rate k were significantly higher in ESBM compared to SBM (*p* < 0.05) (Table 4). During the incubation with pancreatin, SBM and ESBM had the mean N release rates k in AA_LMW of 0.0123 min^−1^ and 0.0733 min^−1^, with D_0_ of 25.24 and 50.15%, and ΔD of 33.38 and 16.42%, respectively (Table 4).

### 3.6 Correlation between protein secondary structures and protein classification as well as *in vitro* protein digestive dynamics

**Figure 3:**
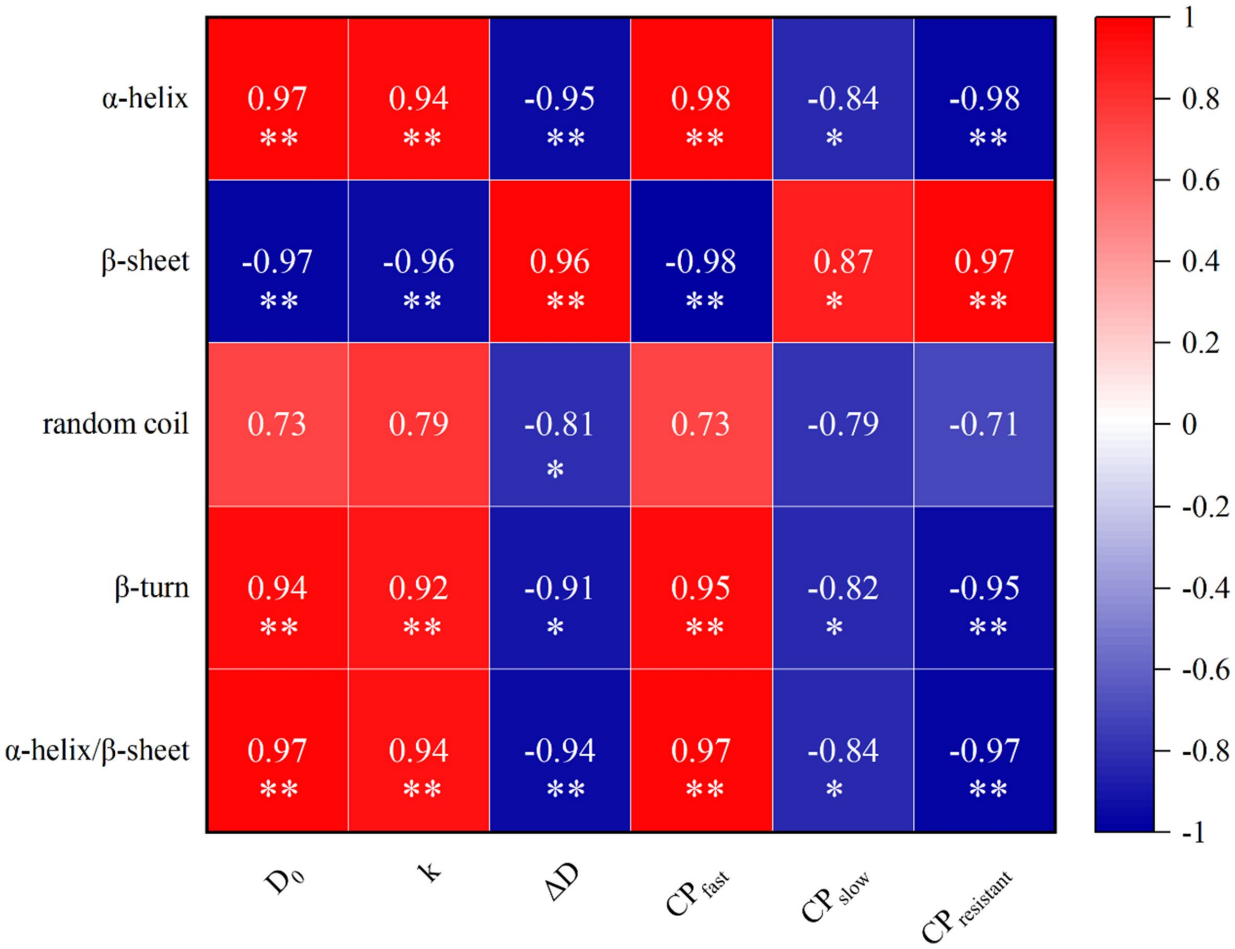
Correlation of protein secondary structure, protein classification and dynamic parameters of protein digestion *in vitro*. Heatmaps depict Pearson’s correlation coefficients. Deep red and dark blue represent stronger correlation coefficients and *p* values. Light red and light blue represent weaker correlation coefficients and *p* values. *p < 0.05; **p < 0.01.

The results of correlation analysis between the content of protein secondary structure, protein classification, and *in vitro* protein digestive dynamics were presented in Figure 3. The content of α-helix was positively correlated with CP_fast_, D_0_, and k (*p* < 0.05), while negatively correlated with CP_slow_, CP_resistant_, and ΔD (*p* < 0.05). Conversely, the β-sheet showed a significant negative correlation with CP_fast_, D_0_, and k (*p* < 0.05) but a positive correlation with CP_slow_, CP_resistant_, and ΔD (*p* < 0.05). The β-turn exhibited a significant positive correlation with CP_fast_, D_0_, and k (*p* < 0.05) while exhibiting a negative correlation with CP_slow_, CP_resistant_, and ΔD (*p* < 0.05). Random coil was significantly negatively correlated with ΔD (*p* < 0.05), while correlations with other components were not significant. Additionally, the ratio of α-helix to β-sheet was significantly positively correlated with CP_fast_, D_0_, and k (*p* < 0.05), and negatively correlated with CP_slow_, CP_resistant_, and ΔD (*p* < 0.05).

## 4 Discussion

Enzymatic hydrolysis processing did not alter the CP, EE, and Ash content of SBM. The variation in the DM content of SBM before and after enzymatic hydrolysis processing could probably be attributed to the drying process that follows wet enzymatic hydrolysis. The microstructure of ingredients significantly impacts their digestibility. Therefore, SEM was employed to examine how the microstructure of SBM changed after enzymatic hydrolysis. Compared to SBM, ESBM exhibited a fibrous surface and a relatively loose internal structure. This might increase the effective contact surface area of ESBM with enzymes during digestion, thereby accelerating hydrolysis. Studies have shown that the extrusion-enzyme hydrolysis treatment significantly altered the conformational and functional properties of soybean protein (31).

The present study investigated the molecular spectral band characteristics of proteins. As shown in Figure 2A, the peaks observed at 3,266–3,282 cm^−1^ correspond to the stretching vibrations of water molecules (O–H) in the SBM and ESBM, indicating a reduction in moisture content in SBM after the enzymatic hydrolysis processing. Amide I, II, and III bands are typically assigned to peaks located at 1,700–1,610 cm^−1^, 1,600–1,500 cm^−1^, and 1,330–1,220 cm^−1^, respectively (32). The position of the Amide I band peaks (1,700–1,600 cm^−1^) changed, indicating changes in the protein molecular structure of SBM after the enzymatic hydrolysis processing. The peaks observed at 1,623.05–1,631.20 cm^−1^ are mainly attributed to the stretching vibrations of the C=O group in the Amide I band. The peaks observed at 1,537.20–1,538.25 cm^−1^ are mainly attributed to the stretching vibrations of the C–N group in the Amide II band. The peaks observed at 1,236.39–1,238.26 cm^−1^ are mainly attributed to the stretching vibrations of the C–N group in the Amide III band. We focused on the protein regions to evaluate the effect of the enzymatic hydrolysis processing on the proteins in SBM. The amide I band is most frequently used for conformational analysis (33). In the present study, after enzymatic hydrolysis of SBM, the α-helix, β-turn, and the ratio of α-helix to β-sheet content were significantly higher and β-sheet content was significantly lower than before. This indicates that the enzymatic processing results in conversion between different protein secondary structures in SBM. It has been reported that fermentation treatment can also cause changes in the protein secondary structure of SBM (34). Different fermentation methods have different effects on the protein secondary structure in corn-soybean meal diets (25).

The *in vitro* method is an effective approach to evaluate the protein quality of ingredients. It has broad application prospects in evaluating the nutritional value of feed. However, there are few studies on the dynamics of *in vitro* protein digestion in pigs and poultry (5, 10, 22, 24, 35, 36). Typically, *in vitro* protein digestion methods were according to a two-step method described by Boisen and Fernández (3) or with modification (3, 10, 36). Nevertheless, there is still no standardized and unified *in vitro* protein digestion method. For example, the incubation time and pH value in the gastric phase vary significantly in different trials (3, 10). It has been reported that low pH (pH 2.0) during *in vitro* gastric digestion may lead to overestimation of the protein digestibility (37), and that pH 3.5 is more likely to mimic the internal environment of the porcine stomach than pH 2.0 (38). Therefore, we used a pH of 3.5 in this trial. Enzymatic hydrolysis processing changed SBM’s protein digestibility and affected N distribution in the different fractions during digestion. Chen et al. (10) observed that N solubility rapidly increased from 47–52% to 80–85% in the first 30 min of the small intestine phase during *in vitro* protein digestion of SBM, and eventually (210 min) reached 91–93%, which is similar to the results in the present study (47, 80, and 82%, respectively). The N present in the AA_LMW of SBM increased from 9–11% to 22–30%, and eventually (210 min) reached 32–44% (10), it was much lower than *in vitro* results in the present study (26, 36, and 54%, respectively). The gaps may be related to the different sources and processing of SBM. In the present study, the changes in N solubility during *in vitro* protein digestion of ESBM were not significant with SBM (except for 60 and 90 min), which is different from the report by Mai Anh Ton et al. (5), probably due to differences in the source and production process of SBM. However, the N present in the AA_LMW of ESBM during *in vitro* digestion was higher than that of SBM at all time points (17–32% higher). This indicates that the increase in the N present in the AA_LMW of ESBM during *in vitro* digestion was mainly due to the hydrolysis of the HMW fraction and not related to changes in the N solubility rate.

During *in vitro* protein digestion, the N content in the HMW fraction of ESBM was lower than that of SBM at all time points. It may be attributed to the internal hydrogen bonds of the protein being disrupted after SBM processing, and the protein was depolymerized to low molecular weight peptides (17, 31). The sequential hydrolysis of intermediate peptides is considered to be the rate-limiting step in protein digestion and absorption during protein digestion in animals (39). We may be able to consider the N present in the HMW as intermediate peptides produced during protein digestion *in vitro* to guide animal production. Studies have shown that free amino acid and small peptide content of SBM increased, antigen degraded, and protein digestibility increased after treatments such as extrusion, enzymatic hydrolysis, and fermentation (16–18, 40).

Enzymatic hydrolysis processing also led to changes in the contents of CP_fast_, CP_slow_, and CP_resistant_ in SBM and ESBM. Some studies have found that slow protein can reduce body weight loss and conserve mobilization of body protein in sows (7), increase litter weight gain in piglets, and improve protein efficiency during lactation (8). Combined with the results from SEM and FTIR, the internal hydrogen bonds of proteins in SBM might be disrupted and the structure altered after enzymatic hydrolysis processing. The increased N release rate and content of AA_LMW during digestion may be related to the structural changes. In animals, proteins need to be hydrolyzed into free amino acids or di-and tri-peptides before they can be transported into enterocytes in the small intestine mucosa for subsequent utilization (41). Therefore, compared with N solubility, the release dynamics of N in the AA_LMW can better reflect the *in vivo* release dynamics of amino acids, di-and tri-peptides from ingredients (42). The dynamic pattern of dietary glucose release could affect the contents and patterns of portal amino acids, thereby enhancing the utilization efficiency of dietary nitrogen (43). Therefore, the release dynamics of N in the AA_LMW can be used to further develop the concept of synchronization release among glucose and AA_LMW, which is likely to improve the utilization efficiency of protein in animals.

The protein secondary structure can partly predict changes in protein classification and *in vitro* protein digestive dynamics. In this study, the content of α-helix or β-turn or the ratio of α-helix to β-sheet was positively correlated with CP_fast_, D_0_, and k. The content of β-sheet was positively correlated with CP_slow_, CP_resistant_, and ΔD. The nutritional value of proteins is closely related to their secondary structure (44). Some studies similar to current results that the content of α-helix or the ratio of α-helix to β-sheet were positively correlated with protein digestibility and amino acid release coefficients, while β-sheets showed a negative correlation with these factors (22, 24). Possibly because β-sheet is rich in hydrogen bonds, which can prevent the digestive enzymatic activities of proteins (22).

## 5 Conclusion

SBM underwent enzymatic hydrolysis processing, resulting in a decrease in the content of β-sheet and an increase in the content of α-helix or β-turn or the ratio of α-helix to β-sheet. Simultaneously, the size of the blocky structure of ESBM became smaller than that of SBM, exhibiting a fibrous surface and a relatively loose internal structure. These alterations might enhance the effective contact surface area of ESBM with enzymes during digestion, thereby accelerating hydrolysis and resulting in a higher CP_fast_ content and release rate of AA_LMW in ESBM compared with SBM. These results provide valuable insights for optimizing the processing methods of protein sources to improve the utilization efficiency of protein in pig feed.

## Data Availability

The original contributions presented in the study are included in the article/supplementary material, further inquiries can be directed to the corresponding authors.
